# Supplementary material for: Public attitudes toward medical waste: Experiences from 141 countries
Source: PLoS One. 2024 May 17;19(5):e0302498. doi: 10.1371/journal.pone.0302498 (PMC11101031; doi:10.1371/journal.pone.0302498)
Supplement: S1 Appendix — (DOCX) [file pone.0302498.s001.docx]

Table S1 The detailed information of public positive attitudes percentage of MW.

| Region | Country ID | Abbreviations | MWP (%) |
| --- | --- | --- | --- |
| Asia | Afghanistan | AFG | 63.4 |
| Asia | Armenia | ARM | 69.3 |
| Asia | Azerbaijan | AZE | 67.2 |
| Asia | Bahrain | BHR | 70.8 |
| Asia | Bangladesh | BGD | 65.2 |
| Asia | Bhutan | BTN | 63.3 |
| Asia | Brunei Darussalam | BRN | 69.1 |
| Asia | China | CHN | 70.1 |
| Asia | Georgia | GEO | 69.8 |
| Asia | India | IND | 65.7 |
| Asia | Indonesia | IDN | 64.1 |
| Asia | Iran (Islamic Republic of) | IRN | 68.2 |
| Asia | Israel | ISR | 71.1 |
| Asia | Japan | JPN | 74.2 |
| Asia | Kazakhstan | KAZ | 67.7 |
| Asia | Kuwait | KWT | 71.4 |
| Asia | Lao People's Democratic Republic | LAO | 64.2 |
| Asia | Lebanon | LBN | 70.2 |
| Asia | Malaysia | MYS | 67.2 |
| Asia | Maldives | MDV | 68.4 |
| Asia | Mongolia | MNG | 65.7 |
| Asia | Oman | OMN | 70.6 |
| Asia | Pakistan | PAK | 63.9 |
| Asia | Philippines | PHL | 64.7 |
| Asia | Qatar | QAT | 70.9 |
| Asia | Saudi Arabia | SAU | 69.2 |
| Asia | Singapore | SGP | 72.2 |
| Asia | Sri Lanka | LKA | 68.4 |
| Asia | Syrian Arab Republic | SYR | 66.8 |
| Asia | Thailand | THA | 68.3 |
| Asia | Timor-Leste | TLS | 64.2 |
| Asia | Türkiye | TUR | 67.9 |
| Asia | United Arab Emirates | ARE | 67.9 |
| Asia | Uzbekistan | UZB | 64.5 |
| Asia | Viet Nam | VNM | 65.2 |
| Africa | Algeria | DZA | 65.3 |
| Africa | Angola | AGO | 66.4 |
| Africa | Benin | BEN | 67.1 |
| Africa | Botswana | BWA | 63.9 |
| Africa | Burkina Faso | BFA | 63.2 |
| Africa | Burundi | BDI | 63.3 |
| Africa | Cameroon | CMR | 64.3 |
| Africa | Central African Republic | CAF | 63.2 |
| Africa | Chad | TCD | 63.1 |
| Africa | Djibouti | DJI | 63.3 |
| Africa | Dominica | DMA | 64.5 |
| Africa | Egypt | EGY | 65.2 |
| Africa | Ethiopia | ETH | 64.2 |
| Africa | Gabon | GAB | 64.4 |
| Africa | Gambia | GMB | 63.2 |
| Africa | Ghana | GHA | 63.2 |
| Africa | Guinea | GIN | 64.4 |
| Africa | Guinea-Bissau | GNB | 64.1 |
| Africa | Hungary | HUN | 69.9 |
| Africa | Kenya | KEN | 63.7 |
| Africa | Lesotho | LSO | 63.7 |
| Africa | Liberia | LBR | 63.3 |
| Africa | Madagascar | MDG | 63.1 |
| Africa | Mali | MLI | 64.1 |
| Africa | Mauritania | MRT | 64.2 |
| Africa | Mauritius | MUS | 68.3 |
| Africa | Morocco | MAR | 67.8 |
| Africa | Mozambique | MOZ | 64.4 |
| Africa | Namibia | NAM | 65.8 |
| Africa | Nepal | NPL | 64.1 |
| Africa | Nigeria | NGA | 64.3 |
| Africa | Rwanda | RWA | 63.2 |
| Africa | Sao Tome and Principe | STP | 64.1 |
| Africa | Senegal | SEN | 63.7 |
| Africa | Seychelles | SYC | 67.4 |
| Africa | Sierra Leone | SLE | 63.5 |
| Africa | South Africa | ZAF | 68.1 |
| Africa | Sudan | SDN | 64.8 |
| Africa | Tanzania (United Republic of) | TZA | 63.4 |
| Africa | Togo | TGO | 63.3 |
| Africa | Tunisia | TUN | 67.3 |
| Africa | Uganda | UGA | 63.2 |
| Africa | United Kingdom | GBR | 72.4 |
| Africa | Zambia | ZMB | 64.1 |
| Africa | Zimbabwe | ZWE | 63.2 |
| North America | Bahamas | BHS | 66.4 |
| North America | Canada | CAN | 74.2 |
| North America | Costa Rica | CRI | 69.2 |
| North America | Cuba | CUB | 70.1 |
| North America | El Salvador | SLV | 66.5 |
| North America | Grenada | GRD | 64.2 |
| North America | Guatemala | GTM | 64.3 |
| North America | Haiti | HTI | 63.8 |
| North America | Honduras | HND | 64.2 |
| North America | Iceland | ISL | 74.4 |
| North America | Mexico | MEX | 66.4 |
| North America | Nicaragua | NIC | 65.2 |
| North America | Panama | PAN | 66.2 |
| North America | Saint Lucia | LCA | 64.2 |
| North America | United States | USA | 72.2 |
| Sorth America | Argentina | ARG | 71.4 |
| Sorth America | Bolivia (Plurinational State of) | BOL | 65.1 |
| Sorth America | Brazil | BRA | 68.3 |
| Sorth America | Chile | CHL | 69.4 |
| Sorth America | Colombia | COL | 68.2 |
| Sorth America | Ecuador | ECU | 65.6 |
| Sorth America | Paraguay | PRY | 66.3 |
| Sorth America | Peru | PER | 67.8 |
| Sorth America | Suriname | SUR | 64.6 |
| Sorth America | Uruguay | URY | 70.2 |
| Europe | Austria | AUT | 73.4 |
| Europe | Belarus | BLR | 70.8 |
| Europe | Belgium | BEL | 73.3 |
| Europe | Bosnia and Herzegovina | BIH | 70.4 |
| Europe | Bulgaria | BGR | 69.2 |
| Europe | Croatia | HRV | 71.4 |
| Europe | Cyprus | CYP | 72.2 |
| Europe | Czechia | CZE | 72.3 |
| Europe | Denmark | DNK | 73.5 |
| Europe | Estonia | EST | 70.7 |
| Europe | Finland | FIN | 73.8 |
| Europe | France | FRA | 73.4 |
| Europe | Germany | DEU | 73.2 |
| Europe | Greece | GRC | 72.2 |
| Europe | Ireland | IRL | 74.3 |
| Europe | Italy | ITA | 73.5 |
| Europe | Latvia | LVA | 69.3 |
| Europe | Lithuania | LTU | 68.9 |
| Europe | Luxembourg | LUX | 74.3 |
| Europe | Malta | MLT | 71.2 |
| Europe | Montenegro | MNE | 70.2 |
| Europe | Netherlands | NLD | 74.4 |
| Europe | Norway | NOR | 74.1 |
| Europe | Poland | POL | 70.4 |
| Europe | Portugal | PRT | 71.3 |
| Europe | Romania | ROU | 70.1 |
| Europe | Russian Federation | RUS | 70.4 |
| Europe | Serbia | SRB | 69.4 |
| Europe | Slovakia | SVK | 70.8 |
| Europe | Slovenia | SVN | 71.2 |
| Europe | Spain | ESP | 72.8 |
| Europe | Sweden | SWE | 73.4 |
| Europe | Switzerland | CHE | 73.9 |
| Europe | Ukraine | UKR | 68.4 |
| Europe | Australia | AUS | 73.9 |
| Europe | New Zealand | NZL | 73.1 |

Table S2 The research minimal database.

| ID | IA | EPI | CHE | HDI | LEB | EYS | HAQ | DM | IAR |
| --- | --- | --- | --- | --- | --- | --- | --- | --- | --- |
| Afghanistan | AFG | 43.60 | 13.24 | 0.48 | 62.00 | 10.30 | 26.00 | 20.06 | 18.40 |
| Albania | ALB | 30.50 |  | 0.80 | 76.50 | 14.40 | 75.00 | 124.78 | 72.24 |
| Algeria | DZA | 47.10 | 6.24 | 0.75 | 76.40 | 14.60 | 63.00 | 15.69 | 62.90 |
| Andorra | AND |  | 6.71 | 0.86 | 80.40 | 13.30 | 95.00 | 200.61 |  |
| Angola | AGO | 52.40 | 2.53 | 0.59 | 61.60 | 12.20 | 33.00 | 5.83 | 36.00 |
| Antigua and Barbuda | ATG | 41.10 | 4.44 | 0.79 | 78.50 | 14.20 | 70.00 | 149.09 |  |
| Argentina | ARG | 48.30 | 9.51 | 0.84 | 75.40 | 17.90 | 68.00 | 287.54 | 85.50 |
| Armenia | ARM | 52.40 | 11.34 | 0.76 | 72.00 | 13.10 | 71.00 | 293.60 | 76.51 |
| Australia | AUS | 60.10 | 9.91 | 0.95 | 84.50 | 21.10 | 96.00 | 60.67 | 89.60 |
| Austria | AUT | 66.50 | 10.43 | 0.92 | 81.60 | 16.00 | 94.00 | 232.00 | 87.53 |
| Azerbaijan | AZE | 38.60 | 4.04 | 0.75 | 69.40 | 13.50 | 66.00 | 97.95 | 84.60 |
| Bahamas | BHS | 30.50 | 5.75 | 0.81 | 71.60 | 12.90 | 66.00 | 211.83 | 87.00 |
| Bahrain | BHR | 58.20 | 4.01 | 0.88 | 78.80 | 16.30 | 72.00 | 89.39 | 99.67 |
| Bangladesh | BGD | 29.60 | 2.48 | 0.66 | 72.40 | 12.40 | 48.00 | 17.85 | 24.80 |
| Barbados | BRB | 35.50 | 6.30 | 0.79 | 77.60 | 15.70 | 71.00 | 194.87 |  |
| Belarus | BLR | 23.10 | 5.86 | 0.81 | 72.40 | 15.20 | 74.00 | 75.33 | 85.09 |
| Belgium | BEL | 51.90 | 10.66 | 0.94 | 81.90 | 19.60 | 93.00 | 285.19 | 91.53 |
| Belize | BLZ | 42.00 | 5.97 | 0.68 | 70.50 | 13.00 | 56.00 | 172.53 |  |
| Benin | BEN | 56.20 | 2.39 | 0.53 | 59.80 | 10.80 | 31.00 | 1.34 | 25.80 |
| Bhutan | BTN | 39.40 | 3.61 | 0.67 | 71.80 | 13.20 | 47.00 | 2.72 | 53.50 |
| Bolivia (Plurinational State of) | BOL | 48.50 | 6.92 | 0.69 | 63.60 | 14.90 | 49.00 | 190.50 | 59.94 |
| Bosnia and Herzegovina | BIH | 50.00 | 9.05 | 0.78 | 75.30 | 13.80 | 72.00 | 492.44 | 73.21 |
| Botswana | BWA | 40.10 | 6.05 | 0.69 | 61.10 | 12.30 | 52.00 | 118.64 | 64.00 |
| Brazil | BRA | 43.60 | 9.59 | 0.75 | 72.80 | 15.60 | 64.00 | 323.24 | 81.34 |
| Brunei Darussalam | BRN | 53.20 | 2.16 | 0.83 | 74.60 | 14.00 | 76.00 | 51.43 | 95.00 |
| Bulgaria | BGR | 45.70 | 7.13 | 0.80 | 71.80 | 13.90 | 77.00 | 543.62 | 70.16 |
| Burkina Faso | BFA | 42.50 | 5.46 | 0.45 | 59.30 | 9.10 | 30.00 | 1.85 | 22.00 |
| Burundi | BDI | 54.00 | 7.99 | 0.43 | 61.70 | 10.70 | 27.00 | 0.32 | 9.40 |
| Cabo Verde | CPV | 44.90 | 4.94 | 0.66 | 74.10 | 12.60 | 35.00 | 18.28 |  |
| Cambodia | KHM | 50.00 | 6.99 | 0.59 | 69.60 | 11.50 | 39.00 | 18.28 |  |
| Cameroon | CMR | 65.90 | 3.60 | 0.58 | 60.30 | 13.10 | 32.00 | 7.29 | 37.80 |
| Canada | CAN | 46.70 | 10.84 | 0.94 | 82.70 | 16.40 | 94.00 | 119.84 | 96.97 |
| Central African Republic | CAF | 28.40 | 7.75 | 0.40 | 53.90 | 8.00 | 19.00 | 2.34 | 10.40 |
| Chad | TCD | 32.80 | 4.35 | 0.39 | 52.50 | 8.00 | 25.00 | 1.17 | 10.40 |
| Chile | CHL | 30.20 | 9.33 | 0.86 | 78.90 | 16.70 | 78.00 | 320.96 | 88.30 |
| China | CHN | 36.90 | 5.35 | 0.77 | 78.20 | 14.20 | 78.00 | 1.10 | 70.40 |
| Colombia | COL | 40.10 | 7.71 | 0.75 | 72.80 | 14.40 | 68.00 | 278.72 | 69.79 |
| Comoros | COM | 42.40 | 5.17 | 0.56 | 63.40 | 11.90 | 33.00 | 18.51 |  |
| Congo | COG |  | 2.08 | 0.57 | 63.50 | 12.30 | 34.00 | 7.00 |  |
| Congo (Democratic Republic of the) | COD |  | 3.54 | 0.48 | 59.20 | 9.80 | 34.00 | 1.61 |  |
| Costa Rica | CRI | 42.50 | 7.27 | 0.81 | 77.00 | 16.50 | 74.00 | 176.16 | 80.53 |
| Côte d'Ivoire | CIV | 41.90 | 3.30 | 0.55 | 58.60 | 10.70 | 27.00 | 3.13 |  |
| Croatia | HRV | 46.30 | 6.98 | 0.86 | 77.60 | 15.10 | 87.00 | 414.08 | 78.32 |
| Cuba | CUB | 47.50 | 11.34 | 0.76 | 73.70 | 14.40 | 76.00 | 75.31 | 74.00 |
| Cyprus | CYP | 58.00 | 7.01 | 0.90 | 81.20 | 15.60 | 90.00 | 98.31 | 90.80 |
| Czechia | CZE | 59.90 | 7.83 | 0.89 | 77.70 | 16.20 | 89.00 | 386.16 | 81.34 |
| Denmark | DNK | 47.50 | 9.96 | 0.95 | 81.40 | 18.70 | 92.00 | 123.59 | 96.55 |
| Djibouti | DJI | 51.20 | 1.80 | 0.51 | 62.30 | 7.40 | 35.00 | 19.13 | 59.00 |
| Dominica | DMA | 77.90 | 5.46 | 0.72 | 72.80 | 13.30 | 61.00 | 102.79 | 76.90 |
| Dominican Republic | DOM | 42.20 | 5.93 | 0.77 | 72.60 | 14.50 | 62.00 | 40.41 |  |
| Ecuador | ECU | 29.60 | 7.82 | 0.74 | 73.70 | 14.60 | 62.00 | 203.50 | 64.60 |
| Egypt | EGY | 46.50 | 4.74 | 0.73 | 70.20 | 13.80 | 58.00 | 24.23 | 71.91 |
| El Salvador | SLV | 35.50 | 7.17 | 0.68 | 70.70 | 12.70 | 63.00 | 65.22 | 54.60 |
| Equatorial Guinea | GNQ | 31.70 | 3.14 | 0.60 | 60.60 | 9.70 | 49.00 | 13.04 |  |
| Eritrea | ERI | 56.60 | 4.46 | 0.49 | 66.50 | 8.10 | 28.00 | 2.90 |  |
| Estonia | EST | 61.40 | 6.73 | 0.89 | 77.10 | 15.90 | 86.00 | 204.52 | 89.06 |
| Eswatini (Kingdom of) | SWZ | 31.80 | 6.78 | 0.60 | 57.10 | 13.70 | 36.00 | 122.57 |  |
| Ethiopia | ETH | 76.50 | 3.24 | 0.50 | 65.00 | 9.70 | 28.00 | 6.59 | 24.00 |
| Fiji | FJI | 31.30 | 3.82 | 0.73 | 67.10 | 14.70 | 48.00 | 97.94 |  |
| Finland | FIN | 62.50 | 9.15 | 0.94 | 82.00 | 19.10 | 96.00 | 112.66 | 92.17 |
| France | FRA | 37.40 | 11.06 | 0.90 | 82.50 | 15.80 | 92.00 | 240.27 | 84.80 |
| Gabon | GAB | 49.70 | 2.77 | 0.71 | 65.80 | 13.00 | 40.00 | 13.75 | 62.00 |
| Gambia | GMB | 77.70 | 3.82 | 0.50 | 62.10 | 9.40 | 36.00 | 15.39 | 36.50 |
| Georgia | GEO | 39.10 | 6.66 | 0.80 | 71.70 | 15.60 | 67.00 | 423.65 | 72.53 |
| Germany | DEU | 27.70 | 11.70 | 0.94 | 80.60 | 17.00 | 92.00 | 181.90 | 89.81 |
| Ghana | GHA | 31.60 | 3.42 | 0.63 | 63.80 | 12.00 | 39.00 | 4.70 | 58.00 |
| Greece | GRC | 36.40 | 7.84 | 0.89 | 80.10 | 20.00 | 90.00 | 319.61 | 78.12 |
| Grenada | GRD | 40.20 | 4.97 | 0.80 | 74.90 | 18.70 | 58.00 | 210.63 | 56.90 |
| Guatemala | GTM | 44.80 | 6.21 | 0.63 | 69.20 | 10.60 | 51.00 | 110.78 | 49.97 |
| Guinea | GIN | 56.20 | 3.98 | 0.47 | 58.90 | 9.80 | 26.00 | 3.46 | 26.00 |
| Guinea-Bissau | GNB | 47.90 | 8.35 | 0.48 | 59.70 | 10.60 | 23.00 | 8.94 | 22.90 |
| Guyana | GUY | 28.00 | 4.93 | 0.71 | 65.70 | 12.50 | 50.00 | 162.86 |  |
| Haiti | HTI | 38.50 | 4.73 | 0.54 | 63.20 | 9.70 | 32.00 | 7.52 | 34.50 |
| Honduras | HND | 36.50 | 7.28 | 0.62 | 70.10 | 10.10 | 47.00 | 111.02 | 42.05 |
| Hong Kong, China (SAR) | HKG |  |  | 0.95 | 85.50 | 17.30 | 82.00 |  | 92.41 |
| Hungary | HUN | 60.20 | 6.35 | 0.85 | 74.50 | 15.00 | 82.00 | 493.56 | 84.77 |
| Iceland | ISL | 26.10 | 8.64 | 0.96 | 82.70 | 19.20 | 97.00 | 62.42 | 99.00 |
| India | IND | 55.10 | 3.01 | 0.63 | 67.20 | 11.90 | 41.00 | 38.32 | 43.00 |
| Indonesia | IDN | 28.20 | 2.90 | 0.71 | 67.60 | 13.70 | 44.00 | 57.86 | 53.73 |
| Iran (Islamic Republic of) | IRN | 18.90 | 6.71 | 0.77 | 73.90 | 14.60 | 72.00 | 172.05 | 84.11 |
| Iraq | IRQ | 57.40 | 4.48 | 0.69 | 70.40 | 12.10 | 51.00 | 63.04 |  |
| Ireland | IRL | 34.50 | 6.68 | 0.95 | 82.00 | 18.90 | 95.00 | 161.77 | 92.00 |
| Israel | ISR | 27.80 | 7.46 | 0.92 | 82.30 | 16.10 | 85.00 | 135.31 | 90.13 |
| Italy | ITA | 62.80 | 8.67 | 0.90 | 82.90 | 16.20 | 95.00 | 294.05 | 70.48 |
| Jamaica | JAM | 48.20 | 6.10 | 0.71 | 70.50 | 13.40 | 62.00 | 112.12 |  |
| Japan | JPN | 57.70 | 10.74 | 0.93 | 84.80 | 15.20 | 94.00 | 36.21 | 90.22 |
| Jordan | JOR | 45.60 | 7.58 | 0.72 | 74.30 | 10.60 | 70.00 | 138.41 |  |
| Kazakhstan | KAZ | 43.60 | 2.79 | 0.81 | 69.40 | 15.80 | 69.00 | 101.47 | 85.94 |
| Kenya | KEN | 57.20 | 4.59 | 0.58 | 61.40 | 10.70 | 39.00 | 10.56 | 29.50 |
| Kiribati | KIR | 40.90 | 10.26 | 0.62 | 67.40 | 11.80 | 26.00 | 11.05 | 38.00 |
| Korea (Republic of) | KOR | 60.00 | 8.16 | 0.93 | 83.70 | 16.50 | 90.00 | 56.14 |  |
| Kuwait | KWT | 30.80 | 5.50 | 0.83 | 78.70 | 15.30 | 81.00 | 60.04 | 99.11 |
| Kyrgyzstan | KGZ | 35.70 | 4.49 | 0.69 | 70.00 | 13.20 | 61.00 | 45.84 |  |
| Lao People's Democratic Republic | LAO | 30.10 | 2.60 | 0.61 | 68.10 | 10.10 | 37.00 | 10.42 | 33.80 |
| Latvia | LVA | 49.00 | 6.58 | 0.86 | 73.60 | 16.20 | 81.00 | 319.48 | 88.90 |
| Lebanon | LBN | 46.90 | 8.65 | 0.71 | 75.00 | 11.30 | 86.00 | 156.59 | 84.10 |
| Lesotho | LSO | 42.40 | 11.27 | 0.51 | 53.10 | 12.00 | 32.00 | 32.96 | 43.00 |
| Liberia | LBR | 30.70 | 8.47 | 0.48 | 60.70 | 10.40 | 32.00 | 5.81 | 25.60 |
| Libya | LBY |  |  | 0.72 | 71.90 | 12.90 | 71.00 | 93.68 |  |
| Liechtenstein | LIE |  |  | 0.94 | 83.30 | 15.20 | 84.00 | 225.50 |  |
| Lithuania | LTU | 32.20 | 7.01 | 0.88 | 73.70 | 16.30 | 80.00 | 343.64 | 83.06 |
| Luxembourg | LUX | 24.90 | 5.37 | 0.93 | 82.60 | 14.40 | 96.00 | 181.48 | 98.82 |
| Madagascar | MDG | 49.40 | 3.69 | 0.50 | 64.50 | 10.10 | 30.00 | 5.09 |  |
| Malawi | MWI | 34.70 | 7.39 | 0.51 | 62.90 | 12.70 | 32.00 | 14.02 |  |
| Malaysia | MYS | 32.30 | 3.83 | 0.80 | 74.90 | 13.30 | 68.00 | 112.49 | 89.56 |
| Maldives | MDV | 55.90 | 8.04 | 0.75 | 79.90 | 12.60 | 70.00 | 56.98 | 62.93 |
| Mali | MLI | 72.30 | 3.89 | 0.43 | 58.90 | 7.40 | 35.00 | 3.66 | 27.40 |
| Malta | MLT | 61.10 | 8.21 | 0.92 | 83.80 | 16.80 | 90.00 | 182.54 | 86.86 |
| Marshall Islands | MHL | 28.40 | 16.34 | 0.64 | 65.30 | 10.20 | 43.00 | 29.10 |  |
| Mauritania | MRT | 42.70 | 3.30 | 0.56 | 64.40 | 9.40 | 41.00 | 21.40 | 40.80 |
| Mauritius | MUS | 28.00 | 6.20 | 0.80 | 73.60 | 15.20 | 69.00 | 80.83 | 64.88 |
| Mexico | MEX | 37.40 | 5.43 | 0.76 | 70.20 | 14.90 | 66.00 | 258.43 | 71.97 |
| Micronesia (Federated States of) | FSM | 45.50 | 11.44 | 0.63 | 70.70 | 11.50 |  | 44.81 |  |
| Moldova (Republic of) | MDA | 36.20 | 6.38 | 0.77 | 68.80 | 14.40 | 67.00 | 294.71 |  |
| Mongolia | MNG | 54.30 | 3.77 | 0.74 | 71.00 | 15.00 | 53.00 | 65.00 | 62.50 |
| Montenegro | MNE | 28.50 | 8.33 | 0.83 | 76.30 | 15.10 | 81.00 | 442.95 | 77.61 |
| Morocco | MAR | 75.20 | 5.31 | 0.68 | 74.00 | 14.20 | 58.00 | 44.10 | 84.12 |
| Mozambique | MOZ | 19.40 | 7.83 | 0.45 | 59.30 | 10.20 | 30.00 | 7.12 | 16.50 |
| Myanmar | MMR | 46.90 | 4.68 | 0.59 | 65.70 | 10.90 | 42.00 |  | 35.10 |
| Namibia | NAM | 29.60 | 8.50 | 0.62 | 59.30 | 11.90 | 45.00 | 160.57 | 41.00 |
| Nepal | NPL | 31.70 | 4.45 | 0.60 | 68.40 | 12.90 | 40.00 | 41.25 | 37.70 |
| Netherlands | NLD | 28.10 | 10.13 | 0.94 | 81.70 | 18.70 | 96.00 | 136.26 | 91.33 |
| New Zealand | NZL | 44.80 | 9.74 | 0.94 | 82.50 | 20.30 | 92.00 | 42.64 | 91.50 |
| Nicaragua | NIC | 40.60 | 8.40 | 0.67 | 73.80 | 12.60 | 61.00 | 3.70 | 45.20 |
| Niger | NER | 35.00 | 5.67 | 0.40 | 61.60 | 7.00 | 28.00 | 1.30 |  |
| Nigeria | NGA | 50.90 | 3.03 | 0.54 | 52.70 | 10.10 | 42.00 | 1.53 | 35.50 |
| North Macedonia | MKD | 37.70 | 7.25 | 0.77 | 73.80 | 13.60 | 79.00 | 458.44 |  |
| Norway | NOR | 28.30 | 10.52 | 0.96 | 83.20 | 18.20 | 97.00 | 76.61 | 97.00 |
| Oman | OMN | 37.70 | 4.07 | 0.82 | 72.50 | 14.60 | 76.00 | 90.63 | 95.23 |
| Pakistan | PAK | 62.60 | 3.38 | 0.54 | 66.10 | 8.70 | 38.00 | 13.86 | 25.00 |
| Palau | PLW |  | 15.15 | 0.77 | 66.00 | 15.80 | 62.00 | 33.32 |  |
| Palestine, State of |  |  |  | 0.72 | 73.50 | 13.40 | 57.00 |  | 74.60 |
| Panama | PAN | 59.30 | 7.58 | 0.81 | 76.20 | 13.10 | 68.00 | 197.11 | 64.25 |
| Papua New Guinea | PNG | 28.30 | 2.30 | 0.56 | 65.40 | 10.40 | 32.00 | 7.47 |  |
| Paraguay | PRY | 56.70 | 7.17 | 0.72 | 70.30 | 13.00 | 57.00 | 274.73 | 73.96 |
| Peru | PER | 30.70 | 5.22 | 0.76 | 72.40 | 15.40 | 64.00 | 657.62 | 65.25 |
| Philippines | PHL | 24.60 | 4.08 | 0.70 | 69.30 | 13.10 | 51.00 | 57.86 | 49.80 |
| Poland | POL | 50.50 | 6.45 | 0.88 | 76.50 | 16.00 | 82.00 | 311.38 | 83.18 |
| Portugal | PRT | 39.80 | 9.53 | 0.87 | 81.00 | 16.90 | 86.00 | 246.34 | 78.26 |
| Qatar | QAT | 28.90 | 2.91 | 0.86 | 79.30 | 12.60 | 82.00 | 23.67 | 99.65 |
| Romania | ROU | 50.60 | 5.74 | 0.82 | 74.20 | 14.20 | 78.00 | 348.85 | 78.46 |
| Russian Federation | RUS | 50.40 | 5.65 | 0.82 | 69.40 | 15.80 | 75.00 | 260.91 | 84.99 |
| Rwanda | RWA | 40.90 | 6.41 | 0.53 | 66.10 | 11.20 | 36.00 | 11.33 | 26.50 |
| Saint Kitts and Nevis | KNA |  | 5.40 | 0.78 | 71.70 | 15.40 | 44.00 | 86.48 |  |
| Saint Lucia | LCA | 33.00 | 4.32 | 0.72 | 71.10 | 12.90 | 63.00 | 220.55 | 53.30 |
| Saint Vincent and the Grenadines | VCT | 56.00 | 4.75 | 0.75 | 69.60 | 14.70 | 57.00 | 104.55 |  |
| Samoa | WSM | 37.50 | 6.36 | 0.71 | 72.80 | 12.40 | 48.00 | 14.79 |  |
| San Marino | SMR |  | 6.39 | 0.85 | 80.90 | 12.30 | 83.00 | 347.69 |  |
| Sao Tome and Principe | STP | 32.80 | 5.53 | 0.62 | 67.60 | 13.40 | 39.00 | 35.13 | 33.00 |
| Saudi Arabia | SAU | 37.90 | 5.69 | 0.88 | 76.90 | 16.10 | 77.00 | 26.93 | 97.86 |
| Senegal | SEN | 27.60 | 4.13 | 0.51 | 67.10 | 9.00 | 31.00 | 11.75 | 42.60 |
| Serbia | SRB | 33.90 | 8.67 | 0.80 | 74.20 | 14.40 | 77.00 | 195.91 | 78.37 |
| Seychelles | SYC | 50.90 | 5.19 | 0.79 | 71.30 | 13.90 | 66.00 | 171.85 | 79.00 |
| Sierra Leone | SLE | 35.00 | 8.75 | 0.48 | 60.10 | 9.60 | 31.00 | 1.58 | 18.00 |
| Singapore | SGP | 32.70 | 4.08 | 0.94 | 82.80 | 16.50 | 91.00 | 28.02 | 92.00 |
| Slovakia | SVK | 40.80 | 6.96 | 0.85 | 74.90 | 14.50 | 83.00 | 377.13 | 89.92 |
| Slovenia | SVN | 43.90 | 8.52 | 0.92 | 80.70 | 17.70 | 91.00 | 329.40 | 86.60 |
| Solomon Islands | SLB | 52.90 | 4.77 | 0.56 | 70.30 | 10.30 | 32.00 | 23.44 |  |
| South Africa | ZAF | 45.90 | 9.11 | 0.71 | 62.30 | 13.60 | 50.00 | 172.40 | 70.00 |
| South Sudan | SSD |  | 6.04 | 0.39 | 55.00 | 5.50 | 27.00 | 1.23 | 6.50 |
| Spain | ESP | 67.30 | 9.13 | 0.91 | 83.00 | 17.90 | 92.00 | 244.83 | 93.21 |
| Sri Lanka | LKA | 72.70 | 4.08 | 0.78 | 76.40 | 14.10 | 71.00 | 78.31 | 35.00 |
| Sudan | SDN | 44.90 | 4.97 | 0.51 | 65.30 | 7.90 | 46.00 | 11.32 | 28.40 |
| Suriname | SUR | 55.60 | 4.57 | 0.73 | 70.30 | 13.00 | 54.00 | 236.09 | 70.06 |
| Sweden | SWE | 28.10 | 9.74 | 0.95 | 83.00 | 19.40 | 95.00 | 202.37 | 94.54 |
| Switzerland | CHE | 34.00 | 10.87 | 0.96 | 84.00 | 16.50 | 96.00 | 162.02 | 94.20 |
| Syrian Arab Republic | SYR | 38.10 | 11.29 | 0.58 | 72.10 | 9.20 | 67.00 | 18.07 | 35.78 |
| Tajikistan | TJK | 37.10 | 7.11 | 0.69 | 71.60 | 11.70 | 52.00 | 1.31 |  |
| Tanzania (United Republic of) | TZA | 37.00 | 3.83 | 0.55 | 66.20 | 9.20 | 34.00 | 1.41 | 22.00 |
| Thailand | THA | 35.10 | 3.79 | 0.80 | 78.70 | 15.90 | 69.00 | 47.17 | 77.84 |
| Timor-Leste | TLS | 43.80 | 7.16 | 0.61 | 67.70 | 12.60 | 43.00 | 10.47 | 29.10 |
| Togo | TGO | 47.80 | 5.73 | 0.54 | 61.60 | 13.00 | 32.00 | 3.47 | 24.00 |
| Tonga | TON | 40.70 | 4.98 | 0.75 | 71.00 | 16.00 | 50.00 | 11.35 |  |
| Trinidad and Tobago | TTO | 26.30 | 7.02 | 0.81 | 73.00 | 14.50 | 64.00 | 302.61 |  |
| Tunisia | TUN | 45.30 | 6.96 | 0.73 | 73.80 | 15.40 | 69.00 | 247.52 | 71.90 |
| Türkiye | TUR | 34.20 | 4.34 | 0.84 | 76.00 | 18.30 | 74.00 | 120.00 | 77.67 |
| Turkmenistan | TKM | 35.80 | 6.57 | 0.75 | 69.30 | 13.20 | 62.00 |  |  |
| Tuvalu | TUV |  | 23.96 | 0.64 | 64.50 | 9.40 | 62.00 |  |  |
| Uganda | UGA | 49.60 | 3.83 | 0.53 | 62.70 | 10.10 | 31.00 | 7.94 | 19.90 |
| Ukraine | UKR | 37.40 | 7.10 | 0.77 | 71.60 | 15.00 | 75.00 | 267.66 | 75.04 |
| United Arab Emirates | ARE | 51.10 | 4.28 | 0.91 | 78.70 | 15.70 | 70.00 | 23.72 | 100.00 |
| United Kingdom | GBR | 38.20 | 10.15 | 0.93 | 80.70 | 17.30 | 90.00 | 307.39 | 94.82 |
| United States | USA | 53.20 | 16.77 | 0.92 | 77.20 | 16.30 | 89.00 | 323.19 | 90.90 |
| Uruguay | URY | 46.40 | 9.35 | 0.81 | 75.40 | 16.80 | 71.00 | 215.94 | 86.10 |
| Uzbekistan | UZB | 20.10 | 5.62 | 0.73 | 70.90 | 12.50 | 63.00 | 4.89 | 71.10 |
| Vanuatu | VUT | 36.90 | 3.36 | 0.61 | 70.40 | 11.50 | 32.00 | 4.78 |  |
| Venezuela (Bolivarian Republic of) | VEN | 36.40 | 5.37 | 0.69 | 70.60 | 12.80 | 68.00 | 20.46 |  |
| Viet Nam | VNM | 37.20 | 5.25 | 0.70 | 73.60 | 13.00 | 60.00 | 44.33 | 70.30 |
| Yemen | YEM |  |  | 0.46 | 63.80 | 9.10 | 43.00 | 7.24 |  |
| Zambia | ZMB | 38.40 | 5.31 | 0.57 | 61.20 | 10.90 | 29.00 | 21.85 | 19.80 |
| Zimbabwe | ZWE | 46.20 | 7.70 | 0.59 | 59.30 | 12.10 | 31.00 | 37.71 | 29.30 |
