# Supplementary material for: Public attitudes toward medical waste: Experiences from 141 countries
Source: PLoS One. 2024 May 17;19(5):e0302498. doi: 10.1371/journal.pone.0302498 (PMC11101031; doi:10.1371/journal.pone.0302498)
Supplement: Attachment — Submitted filename: Response to Reviewers.docx [file pone.0302498.s002.docx]

Dear Reviewer:

I would like to thank you for your detailed revisions of this manuscript, which have improved the quality of this manuscript in many ways and have helped me to advance my academic career and that of my collaborators, for which I am grateful! I have responded to your comments on a one-to-one document as follows:

Question 1:

A question at the very beginning must be how do the major findings relate to how people in more developed nations priorly know about such issues to distinguish them from elsewhere ? It is intuitive that such advanced nations will have folks who are more aware of environmental issues. It is an interesting finding of resilience that can be meaningful for more strategizing for greater social action across nations.

Answer 1:

Thank you for your meaningful review. We agree with you and believe that highlighting how people in more developed nations prior know about such issues to distinguish them from elsewhere is the focus of this study. We adopt the following approach: firstly, we emphasise the importance of revealing that MW public attitudes are more positive in developed countries, which tend to have higher MW emissions and higher amounts of pollution due to MW as shown in lines 51-55; secondly, we highlight the study's "Identifying whether there are significant differences in MW public attitudes between developed and developing countries" as the purpose of the study, as shown in lines 72-73; third, we focus on the relationship between the level of development of a given country and MW attitudes in the " Discussion" section, as shown in lines **345-358**.

Question 2:

Since this is an unfunded study, it reveals that this was significant and commendable work to assemble, retrieve, and analyze the massive data and then write the manuscript. However, a major question: Of those surveyed, what each person's awareness of medical waste and the dangers it may pose what was known beforehand? Was the study intended as an intervention to promote awareness? Is there any indication that this indeed happen?

Answer 2:

Thank you for your meaningful suggestions. Of course, our paper aims to study how to improve public awareness of MW, and we also propose relevant solutions through our cross-sectional analyses, and our study is focusing on public attitudes towards MW. Of course, we understand the importance of randomised controlled trial for the experiment, but we can't obtain the awareness of medical waste that each person possessed before the survey. we just reveal that the public attitude towards MW is related to Risk, Resilience. Environment, and Development variables of interest. However, as we stated above, this is only a cross-sectional study, and we did not impose the exact intervention, and therefore cannot fully causally account for the effect of imposing a given intervention (e.g., improving development, or improving frustration tolerance) on public attitudes toward MW. Our conclusions can only be partially illustrated in the cross-section by analysing the underlying trends in the data based on statistical methods to illustrate how to enhance positive public attitudes towards MW. We clearly understand the importance of RCTs for the scientific validity of experiments, but studies based on sentiment analyses rarely use RCTs, and we have added geographically weighted regressions to traditional sentiment analyses, thus drawing more realistic conclusions. Of course, we would cite the lack of use of RCT as a limitation of this study, as shown in line **433-437**.

Question 3:

Where does medical waste end up in various nations? Can the authors address this from the literature and popular press to query whether it affects public attitudes? The research also did not identify correlates to other pollution causes and can this receive some comment?

Answer 3:

Thank you for your meaningful review. We think that the issue you raise is important and we have added a large amount of literature on the heterogeneity of the way MW is disposed of in different countries, as shown in lines **120-130**. But unfortunately, there is no official document or any literature that quantifies a specific indicator of the way MEDICAL WASTE ENDS UP in different countries, and we have gone through nearly fifty or so related papers, checking the ddocuments of Center for Disease Control and prevention (CDC), available at (<https://www.cdc.gov/datastatistics/index.html>），World Bank 2022 reports, available at ([https://data.worldbank.org/indicator/)](https://data.worldbank.org/indicator/SH.XPD.CHEX.GD.ZS), Environmental Performance Index (EPI) reports(<https://epi.yale.edu/downloads>), United Nations Development Programme, available at ([https://hdr.undp.org/system/files/documents//hdr2020pdf](https://hdr.undp.org/system/files/documents/hdr2020pdf.pdf)), Unfortunately, however, no quantifiable indicators have been found on the way in which medical waste ends up in different countries.

On the other hand, as far as you are concerned, the association with other pollution causes is not the focus of this study. I make the following explanation; because the source of medical pollution is relatively homogeneous, the vast majority of it originates from hospitals and a small portion from households, and medical pollution is hospital-led or government-led in the process of dealing with it, which means that it is detached from the public. This unique characteristic determines that he is not as ubiquitous in everyone's daily life as other causes of pollution, such as air pollution, water pollution, light pollution. So we did not choose to control these causes of pollution. In addition, because of the large number of countries involved in our study, and the difficulty of finding high-quality data on the causes of pollutionof all valid countries, the inclusion of some of the variables could lead to too many missing values and affect the validity of the statistical conclusions.

Question 4:

The research objective number 3 is absolutely key in terms of factors that influence public attitudes. It is noted that the time of the study was during the COVID outbreaks across the world so how might this epidemic heightened the findings in terms of generalized anxiety and concerns about population health? This could bear importantly upon the findings and the conclusions.

Answer 4:

Thank you for your meaningful review. We think the question you raise is important, and we have added information about the temporal trends in public interest in MW during COVID-19 and as an important focus of this study. As shown in lines **279-284 and figure 3**.

Question 5:

As to methodology, this section in particularly needs a little more clarity and shortening. Twitter and crawler mining is an interesting and inexpensive mechanism for research into public views, but what inherent biases might come from these accounts where a large database is needed? Should there be a list of all the nations surveyed and perhaps percentages of the 1.3 million responses, which is somewhat amazing. The tables could receive a little more explanatory commentary for readers to understand better.

Answer 5:

Thank you for your meaningful review. We think the issue you raise is important, and we have removed a large number of unimportant statements in the METHODOLOGY section, from 1,651 words to 1,234 words. Regarding the second issue, inherent biases in the selection of the country sample, we controlled for each country's exposure to MW risk, environment, level of development, resilience, and Internet connectivity in our analyses in the manuscript, as well as using a spatial regression model that geographically weighted all latent variables, and thus can be considered to select a sample of countries with essentially no inherent biases. We account for this phenomenon in the methodology section to ensure that we do not mislead the reader. Regarding the third question, we add an **Appendix with Table S1** that meticulously illustrates the number of tweets about MW in all countries, sentiment attitudes to ensure the credibility of the study.

Question 6:

Finally, English editing is most important where some of the areas are rather tedious to read, but overall it is an important study that revealed cross-country heterogenicity and the potential shaping mechanisms.

Answer 6:

Thank you for your meaningful review. We invite our native English-speaking colleagues to carefully revise the manuscript in order to improve the level of English expression in this manuscript. As shown in the all **“tracking”** in the present manuscript.

Question 7:

In the ending paragraphs, the policy recommendations appear rather broad and could use a bit more specificity for practical responses by policymakers.

Answer 7:

Thank you for your meaningful review. We have rewritten the policy recommendations section to make it more practical. More specifically, we have cited experiences from developed countries such as the United States, Canada, and France, and introduced some relevant MW practices to make the policy recommendations more meaningful. As shown in lines **410-428.**
